# Behavioral recurrence mechanisms of “pain shadow memory” and nursing intervention opportunities in postherpetic neuralgia during the skin lesion regression stage

**DOI:** 10.3389/fmed.2026.1861594

**Published:** 2026-07-24

**Authors:** Guoqin Li, Wuchao Wang, Qinxin Shu, Jian Huang, Yuqiong Yang

**Affiliations:** 1 Department of Pain, Daping Hospital, Army Medical University, Chongqing, China; 2 Department of Nursing, Daping Hospital, Army Medical University, Chongqing, China

**Keywords:** behavioral reproduction, postherpetic neuralgia, nursing pathway, pain memory, regulatory opportunities

## Abstract

**Objective:**

To address clinical nursing challenges associated with postherpetic neuralgia(PHN), including recurrent pain shadow memory (PSM), refractory pain, and marked negative affect, this study assessed the effectiveness of a staged nursing pathway and identified key opportunities for cognitive–sensory–behavioral staged regulation, thereby providing a scientific foundation for improving nursing care for patients with PHN.

**Methods:**

A total of 100 patients diagnosed with PHN between July 2025 and April 2026 were enrolled and assigned to either a control group or an experimental group according to the admission period in a quasi-experimental design. The control group received routine nursing care, such as general nursing (e.g., vital signs, observation of the patient’s condition, and medication administration). The observation group received a step-by-step nursing intervention, in addition to the control group’s treatment, with three steps as follows: cognition correction (correcting irrational cognition about pain), sensory intervention (cold compress treatment combined with sensory desensitization), and behavioral counseling (desensitization language combined with behavior intervention). Results were evaluated with the Pain Shadow Behavior Identification Scale, the Numerical Rating Scale (NRS), and the Hospital Anxiety and Depression Scale (HADS). Data were analyzed using a t-test.

**Results:**

Baseline characteristics were comparable between the two groups (all *p* > 0.05). After the intervention, the experimental group demonstrated significantly greater improvements in all three behavioral dimensions of pain shadow memory, namely situational recurrence, action avoidance, and verbal response, than the control group on both day 4 and day 7. On day 4, the experimental group showed significantly lower scores in situational recurrence (1.70 ± 0.54 vs. 1.96 ± 0.40, *p* < 0.001), action avoidance (1.70 ± 0.61 vs. 2.76 ± 0.43, *p* < 0.05), and verbal response (1.88 ± 0.52 vs. 2.32 ± 0.55, *p* < 0.05). On day 7, the experimental group continued to demonstrate significantly greater improvements in all three dimensions: situational recurrence (0.68 ± 0.65 vs. 1.64 ± 0.48, *p* < 0.005), action avoidance (1.30 ± 0.46 vs. 2.06 ± 0.24, *p* < 0.001), and verbal response (0.94 ± 0.42 vs. 1.72 ± 0.50, *p* < 0.01). The Numerical Rating Scale score was significantly lower in the experimental group than in the control group on day 4 (4.78 ± 0.88 vs. 5.22 ± 0.58, *p* < 0.05) and day 7 (2.56 ± 0.54 vs. 3.82 ± 0.66, *p* < 0.001). Regarding emotional status, the experimental group showed significantly lower scores on both the Hospital Anxiety and Depression Scale-Anxiety subscale (8.52 ± 1.89 vs. 9.88 ± 1.43, *p* < 0.001) and the Hospital Anxiety and Depression Scale-Depression subscale (7.68 ± 1.39 vs. 8.72 ± 1.14, *p* < 0.001) compared with the control group on day 7.

**Conclusion:**

The staged nursing pathway, which integrates sequential cognitive, sensory, and behavioral interventions, significantly reduced the recurrence of pain shadow memory, rapidly relieved pain, and improved anxiety and depression in patients with postherpetic neuralgia. This approach addresses the limitations of conventional nursing care and provides a feasible, effective, and evidence-based strategy for clinical practice.

## Introduction

Herpetic neuralgia (HN), which is caused by reactivation of the varicella-zoster virus (VZV), is a type of neuropathic pain. Its clinical subtypes include postherpetic neuralgia (PHN), which has a prevalence of up to 30–50% among individuals older than 50 years (1), with an incidence that increases with age. It is a chronic pain syndrome with typical features, such as burning pain and electric-shock-like paroxysms, which are commonly associated with the development of so-called “pain shadow memory” (PSM), defined as the consolidation of painful experiences in the central nervous system (CNS) through neuroplastic processes. These processes lead to the formation of conditioned reflex-like memories, including situation association, action avoidance behavior, and verbal response (2).

Recent studies have suggested that pain memory is an important mechanism underlying the transition from acute pain to chronic pain. Pain-related experiences can induce long-term neuroplastic changes in brain regions involved in sensory processing, emotional regulation, and behavioral responses, including the anterior cingulate cortex, the amygdala, the hippocampus, and the prefrontal cortex (3). These changes contribute to the formation and persistence of pain-related memories, which may be reactivated by environmental cues, sensory stimuli, or emotional triggers even after the original nociceptive stimulus has diminished. Emerging evidence indicates that pain memory not only amplifies pain perception but also promotes fear-avoidance behaviors, negative emotional states, and functional impairment. Consequently, the modulation of pain memory has become an increasingly important research focus in chronic pain management and rehabilitation. Although the concept of pain memory has been widely discussed in chronic pain research, studies specifically focusing on pain shadow memory in patients with herpetic neuralgia remain limited. Existing evidence suggests that patients with herpetic neuralgia frequently exhibit fear-related avoidance behaviors, heightened pain vigilance, and persistent negative emotional responses even after the acute phase of the disease (4). These manifestations are considered to be closely associated with pain memory processes. Previous studies have primarily focused on pharmacological treatment, pain control, and psychological support, whereas the behavioral characteristics, recurrence mechanisms, and nursing management of pain shadow memory have received relatively little attention. Therefore, further investigation is needed to better understand the behavioral manifestations of pain shadow memory and to identify effective nursing strategies for its management in patients with postherpetic neuralgia.

In humans, aberrant PSM activity is known to enhance pain sensitivity, promote the consolidation of maladaptive behaviors, and increase the risk of comorbid emotional disorders. The incidence of anxiety and depression in PHN patients was 40–60% higher than that in simple neuralgia patients, severely affecting their day-to-day activities and quality of life (5). Existing clinical nursing interventions in the management of HN are limited to passive treatment that includes monitoring adverse drug reactions and simple skin care, without much focus on the mechanisms underlying PSM formation or their subsequent behavioral repetition (6).

The literature suggests that multiple pathophysiological processes are involved in the generation of PSM, such as the sensitization of the sensory afferent pathway, consolidation of emotional valence within the limbic system, and consolidation of avoidance behavior within the motor cortex. PSM may recur because of environmental triggers, such as contextual (e.g., similar contexts and postures of the body) and sensory impressions (e.g., light touch and temperature changes), resulting in an increased frequency of situational recurrence, intensified avoidance behaviors, and increased negative verbal responses (7). However, there exists no clinical nursing intervention system based on the behavioral identification of PSM. Evidence-based guidelines are lacking with regard to when and how intense the multi-dimensional regulating measures should be, for example, cognitive restructuring, sensory reconditioning, and behavior therapy. Therefore, despite temporary relief from pain symptoms, a majority of patients still suffer recurring pain and psychological distress because PSM persists (8).

To fill these gaps, in this study, we aim to analyze the evolution of some key behavioral dimensions of PSM—situational recurrence, action avoidance, verbal responses, and pain frequency—in PHN patients by developing a behavioral identification scale of PSM, thus explaining the behavioral mechanisms underlying PSM recurrence. In addition, the effect of standard nursing and phased nursing pathways in terms of pain score, mood states, and PSM-related behaviors is compared, with a view toward identifying critical opportunities and optimization avenues for nursing interventions. The results are expected to offer an evidential basis for developing accurate, personalized care plans for patients with PHN.

## Materials and methods

### Study design and participants

Participants were grouped according to the admission period, resulting in a quasi-experimental historical control design. This study employed a quasi-experimental, non-randomized controlled design and was conducted from July 2025 to April 2026. Patients were allocated to either the control group or the experimental group according to their admission period rather than random assignment. The control group consisted of 50 patients admitted between July and November 2025, whereas the experimental group consisted of 50 patients admitted between December 2025 and April 2026. Ethical approval for this study was obtained on December 9, 2025, and all informed consent forms were signed after that date. As the control group received routine care without additional study interventions, the ethics committee granted a waiver of informed consent for these patients; the experimental group, by contrast, all provided written informed consent prior to enrollment.

Patients were eligible for inclusion if they met the following criteria: (1) a diagnosis of PHN conforming to the Chinese Expert Consensus on the Diagnosis and Treatment of Postherpetic Neuralgia (2023 Edition); (2) pain persisting for at least 30 days; (3) at least one pain-associated conditioned behavior based upon the use of the Pain Shadow Behavior Identification Scale, such as pain precipitated by a specific situation, avoiding specific movements, or expressing pain verbally; (4) age ≥50 years; (5) the baseline Numerical Rating Scale(NRS) score of ≥4; (6) able to communicate and cooperate well; and (7) providing written informed consent.

Exclusion criteria were as follows: (1) concurrent severe neuropathic pain from other etiologies, such as diabetic peripheral neuropathy or cancer-related pain, or acute pain conditions, such as postoperative or fracture-related pain; (2) presence of psychiatric disorders, including schizophrenia or bipolar disorder, or severe cognitive impairment (Mini-Mental State Examination score <24); (3) pregnancy or lactation; (4) severe dysfunction of major organs, such as heart failure (New York Heart Association class III–IV) or decompensated cirrhosis; (5) known allergy to any materials used in the nursing interventions, including saline or gauze used in cold therapy; and (6) inability to complete the follow-up assessments.

Baseline demographic and clinical characteristics included age, sex, duration of pain, affected anatomical region, comorbidities, baseline pain intensity, concomitant analgesic medication use, pre-intervention NRS score, and pain memory behavior score were comparable between the two groups, with no statistically significant differences observed between the two groups at baseline (all *p* > 0.05). The study protocol was approved by the institutional ethics committee (No: 2025–383, Approval date: 2025.12.9), and it was performed in accordance with the principles of the Declaration of Helsinki.

**Figure 1:**
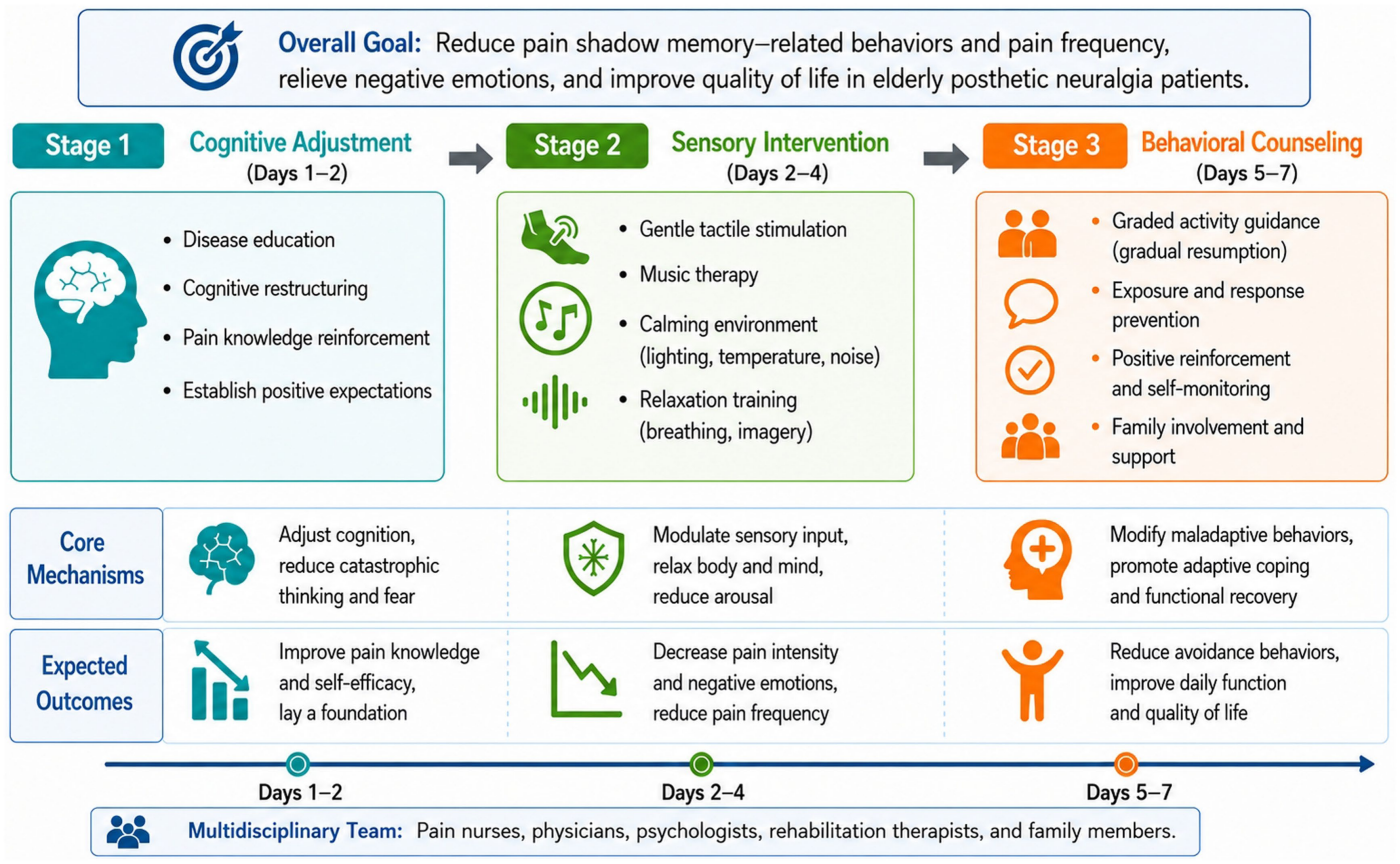
Schematic representation of the staged nursing pathway for patients with postherpetic neuralgia. The intervention consisted of three sequential phases: cognitive adjustment (days 1–2), sensory intervention (days 2–4), and behavioral counseling (days 5–7). The pathway was designed to reduce pain shadow memory-related behaviors and improve pain and emotional outcomes.

Figure 1 illustrates the roadmap for this research.

### Interventions

#### Control group

Patients in the control group received standard nursing care for PHN over a 7-day intervention period. The standard care protocol included the following components: (1) basic nursing: daily skin assessment and care at the painful site to maintain cleanliness and dryness, avoidance of friction and irritation, and monitoring of vital signs, such as body temperature, pulse, respiration, and blood pressure, twice daily; (2) condition observation: documentation of pain episode timing, frequency, and intensity using the NRS; (3) medication management: guidance on the correct administration of analgesics, including pregabalin and gabapentin, according to prescribed dosages and schedules, with monitoring and management of adverse effects such as dizziness, drowsiness, and gastrointestinal discomfort; and (4) routine health education: standardized education sessions conducted at admission and on day 7, covering disease-related knowledge, dietary recommendations (a light diet and avoidance of spicy and irritating foods), and rest guidance (7–8 h of sleep per night and avoidance of overexertion), supplemented with informational brochures.

#### Experimental group

The patients of the experimental group were given the phased nursing pathway on top of the conventional care mentioned above, and the duration of the intervention was 7 days, with an overall daily intervention duration of 60–90 min. The dosage of the interventions was individualized and titrated up depending on patients’ tolerance, based on the principles of low-intensity initiation and step-wise progression; the pathway was provided over a series of three consecutive phases. The staged nursing pathway was developed by the research team based on published evidence regarding pain memory, fear-avoidance behavior, chronic pain management, cognitive-behavioral interventions, sensory desensitization, and rehabilitation nursing. The intervention framework was further refined through multidisciplinary discussions involving pain specialists, nursing experts, and rehabilitation therapists to ensure its clinical feasibility and relevance for patients with herpetic neuralgia. Therefore, the pathway was not entirely self-developed but represented an evidence-informed nursing program adapted to the characteristics of pain shadow memory in postherpetic neuralgia.

##### Stage 1: cognitive adjustment (days 1–2)

In this phase, we used both types of cognitive intervention: individualized focused interaction (20 min a day) and group sessions (40 min each, once a week, with a maximum of 10 participants per group). The content was targeted at informing patients regarding the neurophysiologic background underlying PHN, such as pain sensitization, as well as conditioning for pain memories. Misbeliefs, such as “pain cannot be controlled” or “pain will never go away,” were identified and challenged. Positive expectations about the controllability of pain memory and the efficacy of evidence-based intervention were cultivated through case histories and quotes from recovered patients. Measurable objectives were set in concert with individual patients. The patients received the Pain Memory Cognitive Handbook, consisting of written and pictographic content as well as QR-codes for accessing additional materials, and were asked to read the handbook for 15 min per day, at a consistent time each day, recording variations in their pain-related cognitions.

##### Stage 2: sensory intervention (days 2–4)

Sensory intervention consisted of three parts. First, we conducted sensory desensitization training twice a day, with each session lasting 15 min. We used a graduated tactile desensitization protocol to conduct the training, starting from gentle touch in the perilesional area using a cotton towel and proceeding toward even softer stimuli, such as brushes, silk, and medical cotton swabs. The stimulus intensity was adjusted according to the patients’ comfort without causing pain. Second, local cold compress therapy was applied twice a day, each time for 20 min; a cold compress with normal saline at 15–20 °C was applied to the affected area. The temperature was confirmed with a thermometer before application, and the skin condition and patient comfort were monitored during the process. Third, personalized music relaxation training: the music was chosen based on patients’ preferences, and guided breathing exercises were provided (inhalation for 4 s, breath hold for 2 s, and exhalation for 6 s). We monitored heart rate variability in order to adapt the rhythm and pace of the music and breathing instruction, thereby facilitating the parasympathetic response.

##### Stage 3: behavioral counseling (days 5–7)

Based on the pre-intervention assessment using the Pain Shadow Behavior Identification Scale, behavioral intervention was personalized for each patient. For patients exhibiting situational avoidance behavior, a step-wise procedure was implemented: the treatment started at 5 min, followed by an increase of 5 min per day until reaching a maximum time of 20 min. In the case of individuals who showed avoidance of movement, an incremental exercise program was designed starting at 5 min of walking per day, which increased in steps of 2–3 min/day until reaching 20 min. According to the functionality of joints, low-intensity exercises such as tai chi and Baduanjin were recommended. For patients with vocalized expression of pain, graded language desensitization training was conducted once a day for 15 min. This included a progressive desensitization of the words “pain” or “herpes,” progressing from indirect language to the direct use of these words, with positive statements (e.g., “This pain is manageable”) to mitigate the affective load of the vocal stimuli.

Moreover, we offered education on good sleeping habits in order to treat the sleep problems related to PSM. We used a protocol for sleep treatment: encouraging good sleep hygiene (sleeping from 22:00 to 6:00 with a 30-min nap) and improving sleeping conditions (temperature 22–24 °C, humidity 50–60%, low illumination, and a calm environment). Patients practiced a progressive muscle relaxation exercise 30 min prior to bedtime, contracting muscles for 5 s and relaxing them for 10 s, starting at the feet and working upward, accompanied by soft background music. Each treatment session took 20 min to complete.

Feedback and adjustment behavioral feedback were provided in the form of a 30-min-long, multidimensional feedback session every week, combining patient self-reporting, family reports, and nurses’ observations; training was modified in terms of intensity and time depending on the patient’s tolerance, with a view to maintaining a progressively increasing level of behavioral engagement.

### Intervention fidelity

To ensure consistency of intervention delivery, all nurses involved in the study received standardized training before participant recruitment. The training covered the objectives, procedures, duration, and implementation requirements of each intervention component. A detailed intervention manual was developed and used throughout the study to standardize nursing practices.

During the intervention period, adherence to the protocol was monitored by a senior nurse supervisor through regular observations and weekly reviews of intervention records. Any deviations from the protocol were discussed and corrected promptly. In addition, intervention duration, frequency, and participant compliance were documented using standardized recording forms to ensure consistency across participants.

### Outcome measures

#### Pain frequency associated with pain shadow memory

The frequency of PSM-induced pain was recorded using a pain diary every day in the treatment phase, and patients were asked to record how many times they experienced pain caused by PSM-related factors, such as the occurrence of certain situations or the performance of certain movements. Researchers collected the pain diary at a fixed time every day, verified the completeness of the records, and computed the mean daily rate. Data were collected at pre-intervention (1 day prior to the intervention period), during the intervention (day 4), and after the intervention (day 7).

#### Emotional status

Psychological state: Psychological states were evaluated with the HADS, including the anxiety scale (HADS-A, seven items) and the depression subscale (HADS-D, seven items). All items were assessed using the 4-point Likert-type scale (from 0 = not at all to 3 = almost always). The range of each subscale score was 0–21: 0–7 was regarded as normal, 8–10 as mild, 11–14 as moderate, and 15–21 as severe, with higher scores indicating greater severity of anxiety or depression. The scale was administered by trained expert nurses following standardized instructions. In case of visual impairment and/or difficulty in writing, a face-to-face individual interview was used. Data collection points were pre- and post-intervention (day 7).

#### Behavioral manifestations of pain shadow memory

The behavioral expression of PSM was measured with the Pain Shadow Behavior Identification Scale that assesses three dimensions: situation repetition (reactions related to pain provoked by exposure to a particular situation), action avoidance (purposeful avoidance of actions which might cause pain), and verbal response (expressing verbal worry about or experience of pain). Every dimension was scored according to the frequency of appearance, where 0 = no (0 times/day), 1 = sometimes (1–3 times/day), 2 = often (4–6 times/day), and 3 = very often (> = 7 times/day). A higher score means more severe PSM-related behaviors. The evaluations were carried out individually by two specialized nurses through observing patients’ behavior (three observations per day,10 min each), patient/family communication (2 times per day, 15 min each), and reviewing the pain diary. The inter-rater reliability was good (Kappa = 0.86). The measurements were performed at baseline, day 4, and day 7.

### Pain Shadow Behavior Identification Scale

The Pain Shadow Behavior Identification Scale was developed by the research team based on a review of the literature on pain memory, fear-avoidance behavior, and behavioral manifestations associated with chronic neuropathic pain. The scale was designed as a practical clinical assessment tool to evaluate three core dimensions of pain shadow memory, namely situational recurrence, action avoidance, and verbal response. Each dimension was scored according to the frequency of occurrence, with higher scores indicating more severe pain shadow memory-related behavioral manifestations.

Prior to implementation, the scale content was reviewed by a multidisciplinary panel consisting of pain specialists, nursing experts, and psychologists to ensure clinical relevance and comprehensibility. Inter-rater reliability was assessed independently by two trained nurses and demonstrated good agreement (Kappa = 0.86). As the instrument was developed primarily for exploratory clinical assessment, further psychometric validation, including construct validity, internal consistency, responsiveness, and the determination of clinically meaningful change thresholds, remains warranted in future studies.

### Statistical analysis

Statistical analyses were performed using SPSS version 26.0 (IBM Corp., Armonk, NY, USA). Continuous variables are presented as mean ± standard deviation, and categorical variables are presented as frequency and percentage. Baseline characteristics were compared between groups using independent-samples t-tests or chi-square tests, as appropriate. Bonferroni correction was applied for multiple *post-hoc* comparisons.

For outcomes measured repeatedly at baseline, day 4, and day 7, a two-way repeated-measures analysis of variance was used to examine the effects of group, time, and the group-by-time interaction. When the interaction effect was significant, *post-hoc* comparisons were performed with Bonferroni correction to account for multiple testing. For outcomes measured only at baseline and day 7, analysis of covariance was performed with the baseline value included as a covariate. A two-sided *p*-value of < 0.05 was considered statistically significant.

## Results

**Figure 2:**
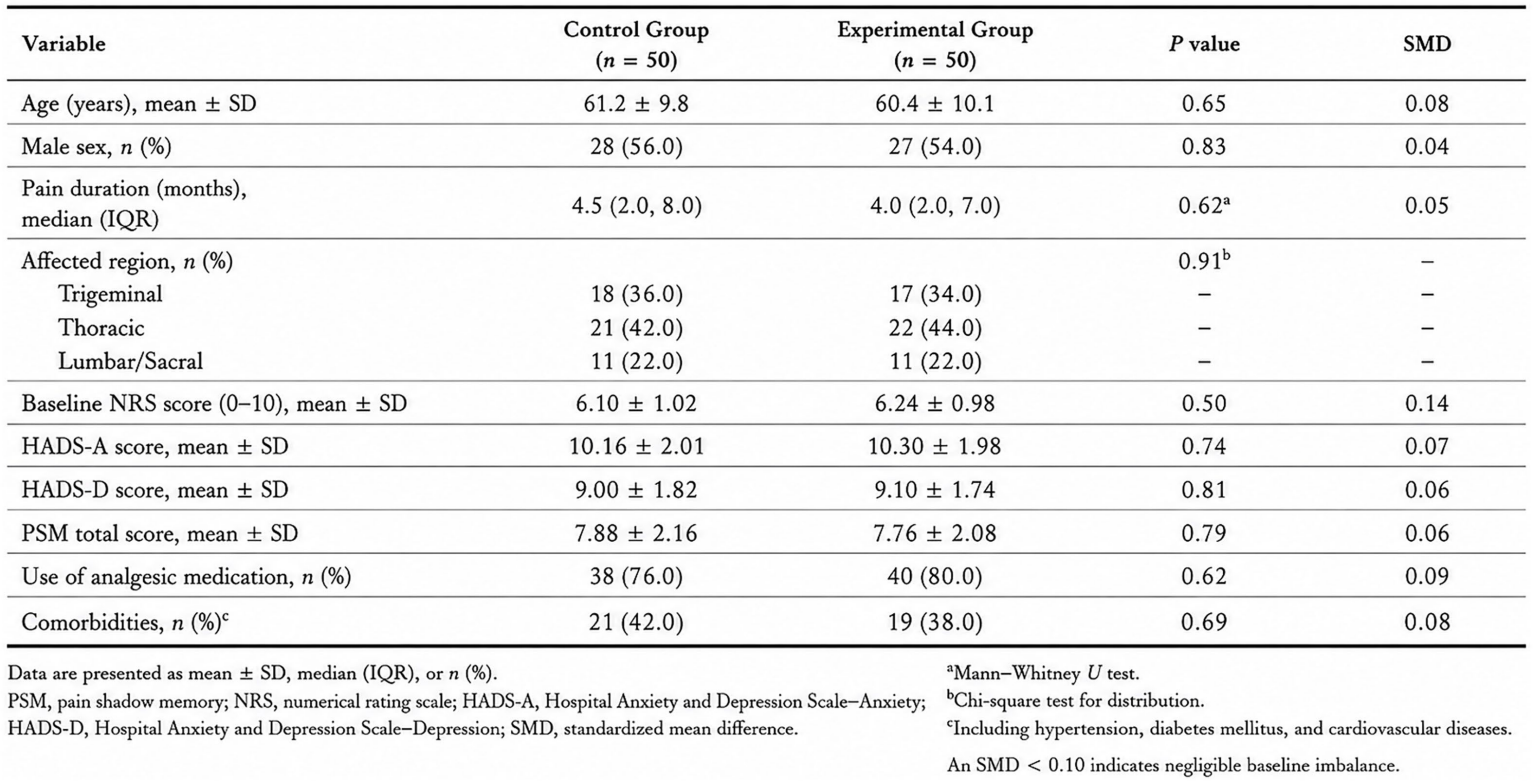
Baseline demographic and clinical characteristics of patients in the control and experimental groups. Data are presented as mean ± standard deviation (SD), median (interquartile range [IQR]), or number (%). Between-group comparisons were performed using independent-samples t tests, Mann–Whitney U tests, or chi-square tests, as appropriate. Standardized mean differences (SMDs) were calculated to assess baseline balance. No statistically significant differences were observed between the two groups at baseline (all *p* > 0.05), and all SMD values were < 0.15, indicating adequate comparability between groups.

The baseline demographic and clinical characteristics of the participants are summarized in Table 1. No significant differences were observed between the control and experimental groups with respect to age, sex distribution, disease duration, affected anatomical region, baseline pain intensity, comorbidities, or analgesic medication use (all *p* > 0.05), indicating good comparability between groups (Figure 2). For repeated outcomes, two-way repeated-measures analysis of variance showed significant group-by-time interaction effects, indicating that changes over time differed between the control and experimental groups. *Post-hoc* analyses with Bonferroni correction further showed that the experimental group had significantly greater improvements than the control group on day 4 and day 7. A total of 100 patients were enrolled in this study, with 50 patients in the control group and 50 in the experimental group. No significant differences were observed between the two groups at baseline in terms of age, sex, disease duration, comorbidities, pre-intervention Numerical Rating Scale score, or pain shadow memory behavioral scores (all *p* > 0.05), indicating that the two groups were comparable. All reported *p*-values for *post-hoc* comparisons were adjusted using the Bonferroni correction.

**Table 1:** Comparison of pain shadow memory-related behavioral dimension scores between the two groups before and after intervention (mean ± SD, points).

Dimension	Group	n	Baseline	Day 4	Day 7
Situational recurrence	Control group	50	2.62 ± 0.49	1.96 ± 0.40	1.64 ± 0.48
	Experimental group	50	2.64 ± 0.48	1.70 ± 0.54	0.68 ± 0.65
	t-value		0.21	2.72	8.35
	*p*-value		>0.05	<0.001	<0.05
Action avoidance	Control group	50	2.94 ± 0.24	2.76 ± 0.43	2.06 ± 0.24
	Experimental group	50	2.90 ± 0.30	1.70 ± 0.61	1.30 ± 0.46
	t-value		0.73	9.98	10.31
	*p*-value		>0.05	<0.05	<0.001
Verbal response	Control group	50	2.64 ± 0.48	2.32 ± 0.55	1.72 ± 0.50
	Experimental group	50	2.60 ± 0.49	1.88 ± 0.52	0.94 ± 0.42
	t-value		0.42	4.10	8.45
	*p*-value		>0.05	<0.05	<0.01

### Pain frequency associated with pain shadow memory

**Figure 3:**
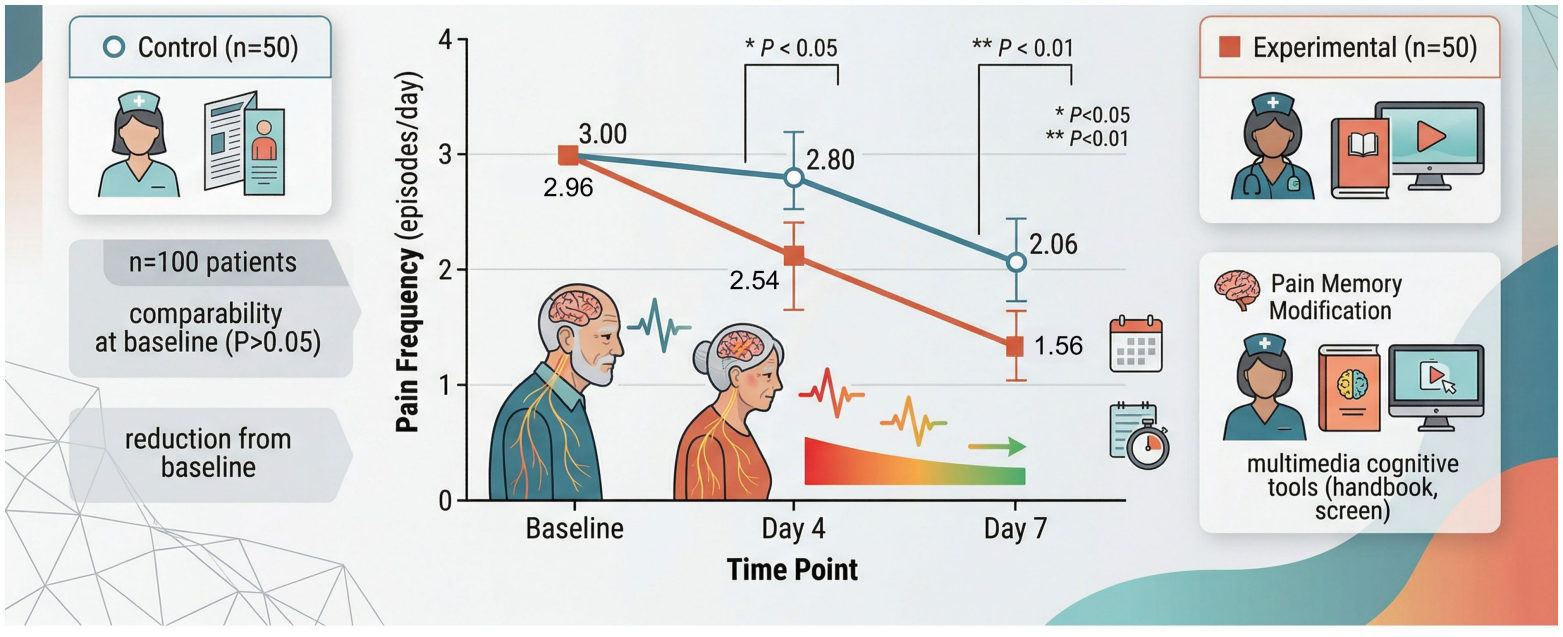
Changes in pain shadow memory-related pain frequency during the intervention period. Data are presented as mean ± standard deviation. Control group (*n* = 50) and experimental group (*n* = 50). Pain frequency was assessed at baseline, day 4, and day 7. Error bars represent standard deviations. Group differences over time were analyzed using repeated-measures analysis of variance followed by Bonferroni-adjusted *post-hoc* comparisons. *p < 0.05; **p < 0.01.

At baseline, the mean daily frequency of pain shadow memory-related pain events was 3.00 ± 0.00 episodes/day in the control group and 2.96 ± 0.20 episodes/day in the experimental group, with no statistically significant difference between groups (*p* > 0.05). On day 4 of the intervention, the pain frequency decreased to 2.80 ± 0.40 episodes/day in the control group and to 2.54 ± 0.50 episodes/day in the experimental group, with the reduction being significantly greater in the experimental group (*p* = 0.0001). On day 7, the pain frequency further decreased to 2.06 ± 0.31 episodes/day in the control group and to 1.56 ± 0.50 episodes/day in the experimental group, and the difference between the two groups remained highly significant (*p* = 0.0000; Figure 3).

### Behavioral manifestations of pain shadow memory

At baseline, there were no statistically significant differences between the two groups in the scores for situational recurrence, action avoidance, or verbal response dimensions (all *p* > 0.05). On day 4 of the intervention, all three dimension scores decreased compared with baseline in both groups, with the experimental group showing significantly greater reductions than the control group (*p* < 0.05). On day 7, the experimental group continued to demonstrate significantly greater improvements in all three dimensions compared with the control group (*p* < 0.05; Table 1).

### Pain intensity

The baseline Numerical Rating Scale scores were comparable between the two groups (*p* > 0.05). On both day 4 and day 7 of the intervention, the Numerical Rating Scale scores decreased significantly from baseline in both groups (*p* < 0.05). Notably, the experimental group had significantly lower Numerical Rating Scale scores than the control group at both time points (*p* < 0.05). The results are shown in Table 2.

**Table 2:** Comparison of Numerical Rating Scale scores between the two groups before and after intervention (mean ± SD, points).

Group	n	Baseline	Day 4	Day 7
Control group	50	7.12 ± 0.56	5.22 ± 0.58	3.82 ± 0.66
Experimental group	50	7.02 ± 0.80	4.78 ± 0.88	2.56 ± 0.54
t-value		0.73	2.93	10.44
*p*-value		>0.05	<0.05	<0.001

### Emotional status

**Figure 4:**
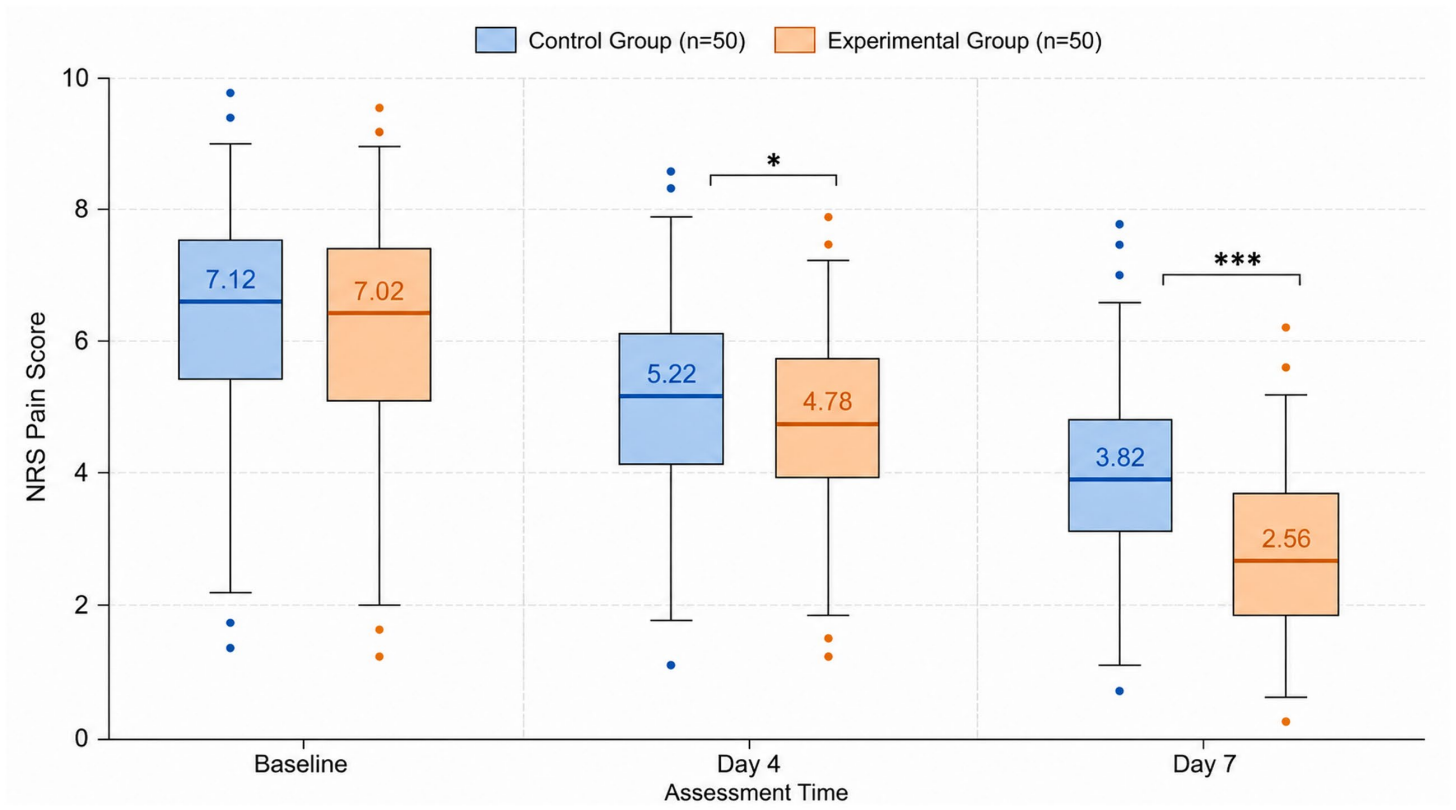
Changes in Numerical Rating Scale (NRS) pain scores during the intervention period in the control and experimental groups. Data are presented as mean ± standard deviation (SD). NRS pain scores were assessed at baseline, day 4, and day 7 after initiation of the intervention. At baseline, no significant difference was observed between the two groups (*p* > 0.05). Both groups demonstrated progressive reductions in pain intensity over time; however, the experimental group showed significantly greater improvement compared with the control group. On day 4, the NRS score was significantly lower in the experimental group than in the control group (4.78 ± 0.88 vs. 5.22 ± 0.58, *p* < 0.05). On day 7, the difference became more pronounced, with the experimental group reporting substantially lower pain scores than the control group (2.56 ± 0.54 vs. 3.82 ± 0.66, *p* < 0.001). Error bars represent standard deviations. NRS, Numerical Rating Scale. *p < 0.05; ***p < 0.001.

At baseline, no significant difference was observed in NRS scores between the two groups (*p* > 0.05). On day 7 of the intervention, both groups showed reductions in NRS scores compared with baseline, with the experimental group demonstrating a significantly greater reduction than the control group (*p* < 0.001). All results are shown in Figure 4.

#### Effect size analysis

**Figure 5:**
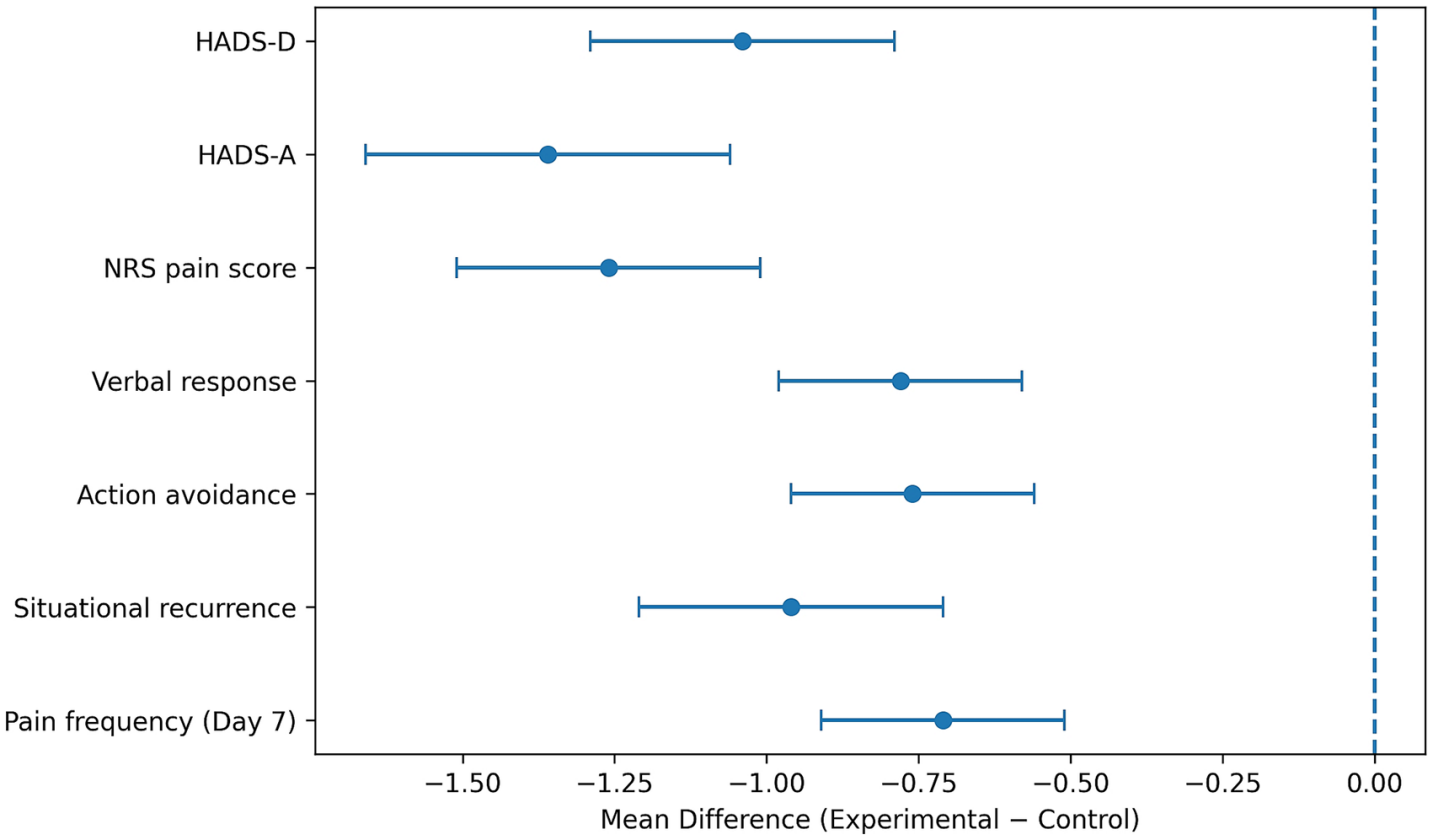
Effect estimates for major clinical outcomes at day 7. Mean differences (experimental group minus control group) and 95% confidence intervals (CIs) are shown for the primary and secondary outcomes assessed at day 7. The vertical dashed line indicates the null effect (mean difference = 0). Values to the left of the null line indicate favorable effects of the staged nursing intervention. Compared with routine nursing care, the staged nursing pathway demonstrated consistent improvements across pain-related outcomes, behavioral manifestations of pain shadow memory, and emotional status. The largest treatment effects were observed for pain intensity (NRS), anxiety (HADS-A), and depression (HADS-D), supporting the clinical effectiveness of the cognitive–sensory–behavioral staged intervention approach.

To facilitate interpretation of the magnitude and direction of treatment effects, between-group mean differences and corresponding 95% confidence intervals (CIs) were calculated for major outcomes at day 7 (Figure 5). Compared with the control group, patients in the experimental group demonstrated lower pain frequency, reduced pain shadow memory-related behavioral manifestations (situational recurrence, action avoidance, and verbal response), lower pain intensity, and lower anxiety and depression scores. All estimated mean differences favored the experimental group, indicating consistent beneficial effects of the staged nursing pathway across pain-related, behavioral, and emotional outcomes.

The largest between-group differences were observed for pain intensity and emotional outcomes (HADS-A and HADS-D), suggesting that the staged nursing intervention may exert synergistic effects on both pain relief and psychological wellbeing (Table 3).

**Table 3:** Comparison of hospital anxiety and depression scale scores between the two groups before and after intervention (mean ± SD, points).

Scale	Group	n	Baseline	Day 7
HADS-A	Control group	50	12.70 ± 1.82	9.88 ± 1.43
	Experimental group	50	13.20 ± 2.33	8.52 ± 1.89
	t-value		1.20	4.04
	*p*-value		>0.05	<0.001
HADS-D	Control group	50	10.80 ± 1.44	8.72 ± 1.14
	Experimental group	50	11.40 ± 2.29	7.68 ± 1.39
	t-value		1.57	4.08
	*p*-value		>0.05	<0.001

## Discussion

This study targeted the fundamental pathological manifestation of “pain shadow memory”(PSM) in PHN patients and established a step-by-step cognitive, sensory, and behavioral intervention. The results showed that this nursing model significantly decreased the frequency of PSM-related pain, improved PSM-related behavior, and decreased the severity of pain and negative mood simultaneously. Our results suggest that the stepwise nursing route is associated with improved outcomes in the nursing care of PHN patients (9).

In patients with PHN, PSM particularly represents neuroplasticity caused by the painful stimulus, in which hypersensitivity to pain and conditioned pain response are present. The traditional nursing care measures mainly provide active treatment for alleviating symptoms, which does not suffice for blocking the activation cycle in PSM (10). The stepwise nursing model used in the current study was based on the sequential interventions of thought restructuring, sensory intervention, and behavioral counseling to control the number of times that PSM-induced pain is triggered.

In this phase of cognitive adjustment, we educated patients about the neurophysiological basis for PSM via individual conversations and special lectures (9), which was effective in correcting misbeliefs, such as a sense of helplessness over their pain, which is consistent with the focus in cognitive restructuring within the Manchester Pain Management Model for managing pain (10). Evidence indicates that negative cognitions in patients with chronic pain can exacerbate pain signals within the CNS, whereas adequate cognitive therapy may mitigate central sensitization, decreasing the number of times with pain (11). In this study, therefore, the decrease in pain frequency after the cognitive intervention in the experimental group is an indication that the cognitive intervention was effective in inhibiting the triggering of PSM.

In the sensory treatment period, tactile gradient therapy and targeted cold compress therapy were used based on the concept of the modulation effect of peripheral sensation on central pain sensitivity (12). Tactile gradient therapy uses different materials and light touch stimulation to gradually increase the sensory threshold at the painful site (13), thereby decreasing the sensitivity of nerve endings (14). The application of cold normal saline compresses to the affected site (at a temperature of 15–20°C) may induce localized vasoconstriction and diminish neurotransmitter release, thus reducing excessive neural excitation (15).

In the behavioral counseling stage, we applied exposure therapy and a progressive plan for activities to break the learned connection between a particular situation and pain (16). Song et al. have found that behavioral interventions grounded in the Cox Health Behavioral Interaction Model have been able to change patients’ behavioral responses by repeating low-dose irradiation, thus lowering the number of conditioned pain episodes (2). In this study, it was found that, after 7 days, the experimental group experienced a decrease in pain frequency, down to 1.56 ± 0.50 episodes/day, which was significantly lower compared with the control group (2.06 ± 0.31 episodes/day; *p* < 0.01). Taken together, these data suggest that the staged nursing pathway can modulate pain frequency through the regulation of the cognitive, sensory, and behavioral domains, thereby blocking the cascade that activates PSM.

The behavioral characteristics of PSM include situation repetition behavior, avoidant behavior, and verbal reaction behavior; these behaviors are not only objective expressions of pain but also important reinforcing factors in the consolidation of PSM (17). Traditional nursing does not have specific intervention measures to address pain behaviors, leading to a failure in the improvement of action avoidance and negative verbal expression (18). In our present research, the stepwise nursing pathway was combined with individualized intervention strategies for each behavior aspect, leading to improved behavioral outcomes.

In terms of situational recurrence, the phased nursing pathway adopted an incremental exposure strategy by progressively increasing the exposure time for each situation, starting at 5 min up to 20 min (19). The use of this “graded exposure” technique not only circumvents high-intensity pain reactions that could be elicited if patients were suddenly exposed to their precipitating factor (20) but also serves to weaken the association between the situation and the pain via repetition (21). The experimental group’s situational recurrence dimension score was 0.68 ± 0.65 at day 7, which was much lower than that of the control group (1.64 ± 0.48, *p* < 0.05).

In terms of avoiding actions, this study developed an incremental activity program based on the concept of “low dose, step-by-step increase,” starting with walking for 5 min per day and progressively increasing to 20 min; it could not only avoid aggravation of pain by excessive activities (22) but also improve patients’ physical function and reduce avoidance behavior through regular activities (23).

For verbal response behaviors, the implemented language desensitization training employed a transitional approach from indirect to direct expression, combined with positive psychological suggestion, to reduce patients’ emotional response intensity to sensitive terms such as “pain” and “herpes” (24). In this study, the verbal response dimension score in the experimental group decreased to 0.94 ± 0.42 on day 7, which was significantly lower than that of the control group (1.72 ± 0.50), indicating that language desensitization training effectively reduces negative verbal expressions and disrupts the vicious cycle of “language reinforcing pain memory.”

Moreover, in the phased nursing intervention, there was a triangulation method including patients’ self-reports, family observation notes, and nurses’ assessments in order to guarantee the objectivity and accuracy of behavior estimation (25). The multiple-source estimation model is consistent with the idea put forward by Guo et al., who noted that continuity of care requires coordination of patient/family and provider efforts (26). It is worth noting that all of the three behavioral dimension scores for the experimental group were significantly lower than those of the control group on day 4, with gradually increasing differences, which indicated a continuously steady effect of phased nursing in improving related behaviors of PSM.

Pain level is related to the patient’s emotional state; severe pain of PHN will induce negative feelings such as anxiety or depression, which in turn are amplified by the central nervous system control and contribute to the vicious circle of “pain-negative emotions” (27). Traditional nursing only provides drug-induced analgesia treatment and basic nursing care without breaking the vicious circle; hence, it has a poor effect on pain relief and little change in mood states (28).

This study achieved the synergistic promotion of pain intensity and emotional status by adopting the step-by-step nursing pathway with a gradual “cognition-sensation-behavior” intervention. Regarding the alleviation of pain, the multi-dimensional intervention of phased nursing had an analgesic effect on both peripheral and central levels (29). On a peripheral level, tactile gradient therapy and local cold compress therapy lowered the sensitivity of nerve terminals and reduced pain signal transmission at the same time. On the central level, cognitive intervention counteracted the amplifying effect of pain signals caused by negative cognition, whereas music relaxation therapy combined with 4–2-6 breathing rhythm guidance inhibited sympathetic nerve excitation and decreased central nervous system pain sensitization (30). The experiment found that the NRS score in the experimental group at 7 days was 2.56± 0.54, which is much lower than that of the control group (3.82 ± 0.66, *p* < 0.001), indicating greater effectiveness than pharmacological nursing alone. This is consistent with Zhang Jing’s opinion of “the effect of multidimensional synergistic nursing is better in terms of analgesia than a single mode.”

Nevertheless, because the present study employed a non-randomized and non-blinded design, the potential influence of performance bias and detection bias cannot be completely excluded. Therefore, future randomized controlled trials with longer follow-up periods are warranted to further confirm the clinical significance and durability of these findings.

### Clinical relevance of the findings

Although statistically significant differences in NRS scores were observed between groups on both day 4 and day 7, the magnitude of improvement differed across time points. The between-group difference on day 4 was relatively small, suggesting that the early clinical impact of the intervention may have been modest. In contrast, the larger difference observed on day 7 indicates a more substantial treatment effect following completion of the staged nursing pathway. These findings suggest that the benefits of cognitive–sensory–behavioral regulation may accumulate over time, resulting in progressive improvements in pain perception, behavioral responses, and emotional status.

Regarding emotional status improvement, the synergistic effect of phased nursing was reflected in three aspects. First, significant relief of pain intensity directly decreased the causes of negative emotions, and a significant drop in pain levels in the experimental group provided a basis for the improvement of the emotional state. Second, cognitive intervention corrected the patient’s negative cognition so as to form the belief that “pain can be controlled,” thus alleviating anxiety and depression caused by pain. Third, personalized training in the behavioral counseling step improved the patient’s behavior, increased their self-efficacy, and reduced negative emotions. In this experiment, on day 7 of the treatment, the experimental group had scores of 8.52 ± 1.89 in the anxiety dimension and 7.68 ± 1.39 in the depression dimension, The anxiety and depression scores of the experimental group were significantly lower than those of the control group. It suggests that phased nursing can break the “pain-negative emotions” vicious circle. Herpes zoster-associated neuralgia imposes a substantial clinical burden, with growing evidence highlighting the complexity of its management and the need for multimodal intervention strategies to reduce recurrence and improve long-term outcomes (31, 32).

Meanwhile, the individualized intervention of phased nursing is another important reason for reaching the synergistic effect. In this research, nurses made an individualized care plan according to the evaluation of the Pain Shadow Behavior Identification Scale and customized relaxation music based on patients’ preferred music genres, which made this type of intervention more suitable for individual needs and improved its efficacy. However, this study has several limitations, including the relatively small sample size, short follow-up period, and the use of a newly developed Pain Shadow Behavior Identification Scale that has not yet undergone comprehensive psychometric validation. In addition, because participants were allocated according to the admission period rather than randomization, the possibility of temporal confounding and selection bias cannot be completely excluded. Future studies should increase the sample size, extend the follow-up period, and further evaluate the long-term effectiveness of the staged nursing pathway. Due to non-random allocation based on admission period, temporal confounding and selection bias cannot be excluded. The absence of blinding may introduce performance and detection bias, particularly for subjective outcomes. The scale is an exploratory assessment tool and has not yet undergone full psychometric validation.

## Conclusion

Behavioral recurrence of “pain shadow memory” in herpetic neuralgia is a consequence of a multilevel interaction between cognition, sensation, and behavior. The staged nursing process, through the gradual application of psychological reformation, sensory stimulation, and behavioral education, effectively reduces the incidence of PSM-associated pain among PHN patients and alleviates pain-associated behaviors, leading to the combined improvement of pain intensity and mood status. This represents a scientific and effective nursing model that provides an important reference for the clinical development of precise and individualized nursing strategies for postherpetic neuralgia.

## Data Availability

The original contributions presented in the study are included in the article/supplementary material; further inquiries can be directed to the corresponding author.
